# Plants reshape protoxylem through tubulin adjustment

**DOI:** 10.1093/plphys/kiae376

**Published:** 2024-07-19

**Authors:** Yang Shao, Jiaqi Sun

**Affiliations:** The Key Laboratory of Plant Development and Environmental Adaptation Biology, Ministry of Education, School of Life Sciences, Shandong University, Qingdao 266237, Shandong, China; Assistant Features Editor, Plant Physiology, American Society of Plant Biologists; The Key Laboratory of Plant Development and Environmental Adaptation Biology, Ministry of Education, School of Life Sciences, Shandong University, Qingdao 266237, Shandong, China

Vascular bundles are integral components of the stele in higher plants, primarily responsible for the conduction of water, minerals, and nutrients (De Rybel et al. 2016). These bundles typically comprise both xylem and phloem tissues arranged together. In the young stems of both dicotyledonous (dicots) and monocotyledonous (monocots) plants, the xylem within the vascular bundles differentiates into 2 types: protoxylem and metaxylem. This differentiation is based on the structural and functional properties of their constituent elements. Protoxylem vessels are among the first xylem elements to differentiate and become functional in young tissues. They provide an immediate pathway for the conduction of water and dissolved minerals from the roots to the growing regions of the plant. This early transport system is essential for sustaining the initial phases of growth before the formation of more mature xylem elements.

It has long been established that the arrangement of cortical microtubules dictates the deposition of secondary cell walls by guiding the trajectory of cellulose synthase complexes (CSCs) (McFarlane et al. 2014; Higa et al. 2024). Cellulose microfibrils are synthesized at the outer surface of the plasma membrane by CSC proteins embedded within the membrane. Microtubules, which are cytoskeletal filaments, are composed of heterodimers of globular α-tubulin and β-tubulin molecules. The assembly and dynamics of these microtubules can be altered by modulating the action of tubulin dimers through post-translational modifications or interactions with microtubule-associated proteins (MAPs) (Janke and Magiera 2020). This modulation is crucial for controlling how and where the cellulose microfibrils are deposited, ultimately influencing the structure and function of the cell wall.

The pattern of wall thickening in protoxylem includes annular and spiral configurations. Annular thickenings form ring-like structures around the cell wall, while spiral thickenings create coiled, spring-like bands of secondary wall material (Pesquet et al. 2011). These patterns have traditionally been thought to facilitate the stretching of vessels, allowing them to elongate as surrounding tissues grow. However, empirical evidence supporting the mechanical or functional equivalence between annular and spiral patterns is still lacking.

In this issue of *Plant Physiology*, Huang et al. (2024) identified the gene *Drought-overly-sensitive 1* (*Dos1*) as being crucial for drought sensitivity through a screening of an ethyl methane sulfonate mutant library in the *Zea mays* cv. “B73” genetic background. Homozygous *Dos1* mutants exhibited a pronounced wilting phenotype in the upper, developing leaves, even under well-watered conditions. These mutants failed to reach the reproductive stage due to arrested growth at the seedling stage. Notably, the wilting leaves of homozygous seedlings remained tightly wrapped, forming a pipette-like structure, as they did not unfold properly. In contrast, heterozygous *Dos1*/+ mutants displayed only mild phenotypes, with observed wilting phenotypes primarily in the young leaves. Despite sophisticated analyses of root and leaf defects, neither was identified as the primary cause for the reduced water accumulation in the mutant leaves that leads to the wilting phenotype.

To further investigate the underlying cause of the wilting phenotype in *Dos1* mutants, detailed examination of the xylem structures was performed using scanning electron microscopy (SEM). In wild-type (WT) plants, protoxylem vessels exhibited the typical annular thickenings. In contrast, *Dos1* mutants displayed markedly different spiral patterns in their xylem. These spiral thickenings frequently detached from the primary walls, undermining the structural support provided to the vessel walls. Moreover, the mutant vessels were visibly narrower than those in WT plants, which may further impair their ability to conduct water effectively and contribute to the wilting phenotype observed in *Dos1* mutants. Supporting this notion, evaluations of sap flow in the stems, water flow within young seedlings, and the use of safranin dye as a water transport tracer demonstrated that water flow in the mutants was significantly slower than in WT plants. These findings suggest that the structural abnormalities in the protoxylem vessels of *Dos1* mutants directly impact their water conduction efficiency, leading to the wilting phenotype.

A point mutation responsible for the *Dos1* mutant was identified in the *α-TUBULIN4* gene (B73: Zm00001d013367), resulting in a single amino acid alteration at position 196 (E196 K). *DOS1* was highly expressed in the xylem, consistent with its essential role in xylem development. Interestingly, the *Dos1* mutation likely has a semi-dominant effect, as the expression of *Dos1* mutant driven by its native promoter in WT background, the *Dos1* transgenic lines remarkably mimicked the phenotype of the *Dos1* heterozygote mutant, exhibiting dramatically altered spiral patterns.

To analyze the dominant effects of the *Dos1* mutation on microtubule distribution, the ectopic VASCULAR-RELATED NAC-DOMAIN 7 (VND7) expression system was utilized. VND7 is a key regulator of xylem vessel differentiation. By ectopically expressing VND7, hypocotyl cells can be induced to differentiate into protoxylem vessel cells, facilitating the study of microtubule array dynamics and secondary cell wall formation (Yamaguchi et al. 2010).

In VND7-expressing plants also expressing GFP-Dos1, the angles between the microtubule arrays and the longitudinal axes of the vessels were significantly greater than in plants expressing GFP-DOS1. This increase in angles of microtubules is consistent with the observed shift from annular to spiral patterns of cell wall thickening in maize *Dos1* mutants. Thus, in the presence of the *Dos1* mutation, the orientation of the microtubule arrays is altered, which likely leads to the formation of abnormal spiral bands in the secondary wall thickenings, and reduced inner diameter of the protoxylem vessels and the water transport efficiency.

**Figure 1.**
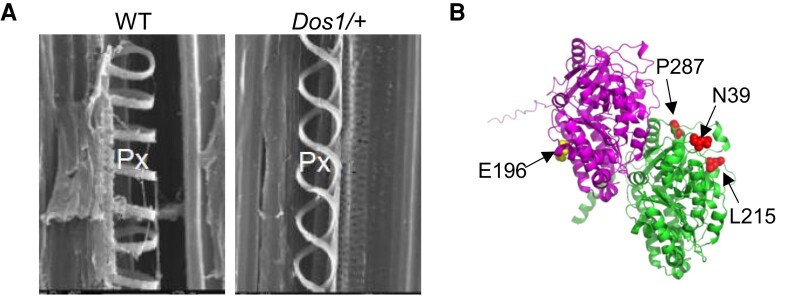
Tubulin variations alter protoxylem vessel patterns. **A)** Shift in protoxylem vessel patterns due to *Dos1* mutation. The *Dos1* mutation causes a dramatic shift in the protoxylem (Px) vessel wall thickening from annular to spiral patterns. This structural change significantly impacts the water conduction rate within the vessels. (Figure adapted from Huang et al. 2024, Fig.). **B)** Tubulin dimer structure model. The protein sequence of DOS1 (α-tubulin 4, Zm00001d013367) was sourced from the Maize Genetics and Genomics Database and WI2 (β-tubulin 6) sequence was from NCBI (XP_008673429.1). The structural model was generated using AlphaFold 3. DOS1 is depicted in magenta and WI2 in green. The *Dos1* mutation position is highlighted by yellow sphere and the mutation positions for WI2 are highlighted by red spheres.

The study by Huang et al. (2024) provides evidence supporting the theory that protoxylem vessels are passively stretched by the growth of surrounding tissues (Karam 2005). According to this theory, the diameters of spiral wall thickenings may shrink when they are passively stretched. These wall thickenings provide structural support when they are pressed against the inner side of the vessel wall. However, if the diameter of the spiral secondary wall decreases and it detaches from the primary cell wall, this can lead to a reduction in structural support and impede water flow (Fig. 1). This theory aligns with the observations from SEM and X-ray micro-computed tomography data. These data show that annular wall thickenings do not experience such mechanical losses of support. When vessels with annular wall thickenings stretch, the ring-shaped thickenings separate, but their diameters remain unchanged, thereby continuing to support the vessel walls effectively.

Despite being more prone to collapse than the annular thickenings, these spiral thickenings are observed across a wide range of plant groups, indicating their significant role in the overall adaptability and functionality of plants. Spiral wall thickenings provide several advantages. For instance, the high flexibility and stretchability of spiral thickenings allow them to accommodate rapid plant growth. This flexibility is crucial during the early developmental stages, when tissues are rapidly expanding. Additionally, spiral thickenings confer resilience against mechanical stresses, such as strong winds or physical disturbances, by allowing the xylem vessels to bend and flex without tearing or collapsing. This structural adaptability supports the integrity and continuous function of the plant's vascular system, making spiral thickenings a valuable feature in diverse ecological contexts (Bailey 1953).

Furthermore, the work by Huang et al. (2024) indicates that a point mutation in the tubulin gene can dramatically alter microtubule alignment, thereby shifting the wall thickening patterns in maize protoxylem vessels. Interestingly, they also characterized a mutant obtained from the Maize Genetic Database with a similar wilting phenotype, *Wilted 2* (*ZmWi2*). Huang et al. (2024) found that *ZmWi2* has a point mutation in a β-tubulin subunit and shows a similar shift towards spiral-shaped secondary wall thickenings, further supporting the role of tubulin dimers in the orientation of the wall thickenings. These results suggest that plants might use changes in their microtubule components, such as tubulins or MAPs, as an evolutionary strategy to adapt their xylem structure to diverse habitats. By adjusting these “microtubule bricks” (Fig. 1), plants can potentially modify their xylem architecture to better withstand different environmental conditions. This understanding opens up the possibility for scientists to leverage this mechanism to enhance crop adaptation to various environmental stresses. By manipulating tubulins or MAPs, it may be possible to engineer crops with xylem structures optimized for improved resilience and efficiency in water and nutrient transport under challenging conditions.
